# Towards Reduction or Substitution of Cytotoxic DMSO in Biobanking of Functional Bioengineered Megakaryocytes

**DOI:** 10.3390/ijms21207654

**Published:** 2020-10-16

**Authors:** Denys Pogozhykh, Dorothee Eicke, Oleksandr Gryshkov, Willem F. Wolkers, Kai Schulze, Carlos A. Guzmán, Rainer Blasczyk, Constança Figueiredo

**Affiliations:** 1 Institute of Transfusion Medicine and Transplant Engineering, Hannover Medical School, 30625 Hannover, Germany; Dorothee.Eicke@gmx.de (D.E.); Blasczyk.Rainer@mh-hannover.de (R.B.); 2 Institute for Multiphase Processes, Leibniz Universität Hannover, 30823 Garbsen, Germany; Gryshkov@imp.uni-hannover.de; 3 Unit for Reproductive Medicine, University of Veterinary Medicine Hannover, 30559 Hannover, Germany; Willem.Frederik.Wolkers@tiho-hannover.de; 4 Department of Vaccinology and Applied Microbiology, Helmholtz Centre for Infection Research, 38124 Braunschweig, Germany; Kai.Schulze@helmholtz-hzi.de (K.S.); CarlosAlberto.Guzman@helmholtz-hzi.de (C.A.G.)

**Keywords:** megakaryocytes, platelets, induced pluripotent stem cells (iPSC), biobanking, cytotoxicity, mouse model, transfusion, dimethyl sulfoxide, propane-1,2-diol, ethylene glycol

## Abstract

Donor platelet transfusion is currently the only efficient treatment of life-threatening thrombocytopenia, but it is highly challenged by immunological, quality, and contamination issues, as well as short shelf life of the donor material. Ex vivo produced megakaryocytes and platelets represent a promising alternative strategy to the conventional platelet transfusion. However, practical implementation of such strategy demands availability of reliable biobanking techniques, which would permit eliminating continuous cell culture maintenance, ensure time for quality testing, enable stock management and logistics, as well as availability in a ready-to-use manner. At the same time, protocols applying DMSO-based cryopreservation media were associated with increased risks of adverse long-term side effects after patient use. Here, we show the possibility to develop cryopreservation techniques for iPSC-derived megakaryocytes under defined xeno-free conditions with significant reduction or complete elimination of DMSO. Comprehensive phenotypic and functional in vitro characterization of megakaryocytes has been performed before and after cryopreservation. Megakaryocytes cryopreserved DMSO-free, or using low DMSO concentrations, showed the capability to produce platelets in vivo after transfusion in a mouse model. These findings propose biobanking approaches essential for development of megakaryocyte-based replacement and regenerative therapies.

## 1. Introduction

In vivo, platelets (PLTs) are produced by megakaryocytes (MK) in the bone marrow and circulate in the blood stream. PLTs play key roles in hemostasis, modulation of inflammatory processes, and wound healing [1,2]. Severe thrombocytopenia may result in life-threatening complications and is often associated with a range of clinical conditions, such as oncological pathologies, septic reactions, acute blood loss, and genetic abnormalities [1,3,4,5]. Currently, efficient handling of severe thrombocytopenia cases may be only achieved by immediate transfusion of the donor PLTs [6]. However, PLT transfusion therapies are compromised by a large number of hurdles associated with immunological parameters, insufficient availability, costly and complicated donation procedures, quality and contamination issues, as well as the short shelf life of the donor material [7,8,9,10,11]. Moreover, pooled PLT concentrates rapidly lose their functional clotting activity required for clinical application upon cold storage [12]. 

In order to overcome the complete dependence on donor blood PLTs for transfusion therapy, current research is focusing on the establishment of robust biotechnological platforms to generate clinically relevant numbers of MK/PLT populations ex vivo [2,7,8,13]. Recent progress in induced pluripotent stem cell (iPSC) technologies and bioreactor systems shows great potential for generation of bioengineered MKs in a large scale [7,13,14,15,16,17,18,19].

Due to the challenges with PLT handling, availability, and storage, significant effort is aimed today at development of alternative approaches, where ex vivo-derived MKs show the capability to release PLTs to blood circulation after transfusion [20,21,22]. 

However, practical implementation in clinics requires readily available off-the-shelf MK supply with high quality and in sufficient numbers. This inevitably faces the necessity of elaboration of adequate biobanking technologies. Cryopreservation is the only existing approach for the long-term storage of cells that enables complete recovery of their vital and functional parameters [23]. Yet, differences in the sensitivity of various cell types and subpopulations to cryopreservation procedures results in highly variable survival rates after thawing [23,24]. Multiple factors that influence the success and outcome of cryopreservation include optimization of cooling and thawing rates, the choice of cryoprotective agents (CPAs) and their concentration, pre-incubation period, thawing procedures and recovery steps [23,25,26]. Several cases of successful cryopreservation of iPSC-derived MKs have been reported, while others report high sensitivity to subzero temperatures and low survival rates [27,28,29,30]. Nevertheless, most of the protocols are based on the application of dimethyl sulfoxide (DMSO) as a CPA. Although the application of 10% DMSO solutions is a golden standard in many methods of cryopreservation, it is known that DMSO is highly cytotoxic and has long-term direct and latent negative side effects [31,32,33,34,35,36]. Thus, several research groups aim at the reduction or complete elimination and substitution of DMSO and serum in cryopreservation techniques intended for application in clinically relevant biobanking [30,34,35,36,37,38,39]. In particular, propane-1,2-diol (PD) and ethylene glycol (EG) are being investigated on variety of cell types as penetrating CPAs alternative to DMSO [38,39,40,41,42,43,44,45,46,47,48,49].

As PLTs appear to be highly sensitive to a wide range of manipulations, including cryopreservation procedures [48,50,51], in this work we focus on cryopreservation of MKs as PLT precursors. The objective of this research is to develop efficient and reproducible biobanking protocols that ensure MK viability and functionality after low temperature storage with reduced content of DMSO, as well as to study the effects of EG and PD as potential DMSO substitutes. Such protocols should aim at reduction or elimination of DMSO-associated risks, be compatible with requirements of good manufacturing practices (GMP) and deliver the possibility of preservation of cells in sufficient amounts for transfusion purposes, where large numbers of MKs/PLTs are required. Accomplishment of these requirements for biobanking of bioengineered MKs/PLTs will lead to the desired progress in this field towards perspectives of clinical application. 

In previous studies, we have shown the possibility to differentiate iPSCs into functional MKs capable of PLT production in a large-scale under defined xeno-free conditions [14,19]. In this work we perform comprehensive analysis of survival, morphologic, structural, and functional properties of iPSC-derived MKs and PLTs before and after cryopreservation procedures in order to define the feasibility to develop efficient biobanking technologies.

As integrin-β3 (CD61), integrin alpha-IIb (CD41), and platelet glycoprotein IX (CD42a) are among the key players that determine the MK efficiency to produce functional PLTs [52], we evaluate the presence of this markers before and after cryopreservation.

During the maturation, MKs undergo endomitosis resulting in polyploid cells [53]. Increase in DNA content is associated with the capacity of MKs to form proplatelets (proPLTs) and release functional PLTs. Therefore, in the frame of this work it was necessary to evaluate, whether the studied in vitro iPSC-derived MKs are polyploid and if this feature is retained in the cell population after freezing. 

In vivo MK cells are localized in the bone marrow and extend long cytoplasmic protrusions, called proPLTs, through the pores of the sinusoidal blood vessels into the circulation [53]. In this study we assess the capability of iPSC-derived MKs to proPLT formation before and after the impact of subzero temperatures under defined conditions. 

PLTs recognize injuries and participate in blood coagulation, to prevent blood loss and facilitate the healing process. Blood clotting is a complex process, in which different factors interact. Thrombin, collagen, and ADP contribute to the PLT activation at the sites of vascular injury [54]. Adherent PLTs undergo cytoskeleton reorganization to fully spread. Similarly, in vitro, when functional PLTs are exposed to fibrinogen and activated with physiological stimulants, such as ADP and thrombin, they adhere to fibrinogen and spread along the surface [55]. Hence, this important functional parameter is evaluated in the present study.

At the same time, while results and parameters analyzed with in vitro studies provide the essential information for the estimation of proposed hypothesis, overlooking fundamental differences between the in vitro and in vivo models may result in serious misleading, especially in the areas with potential clinical application. Therefore, to support the results of in vitro observations we perform transfusion of iPSC-derived MKs before and after cryopreservation procedures in a mouse model.

## 2. Results

### 2.1. Survival and Phenotypic Characterization of MKs after Cryopreservation

**Figure 1:**
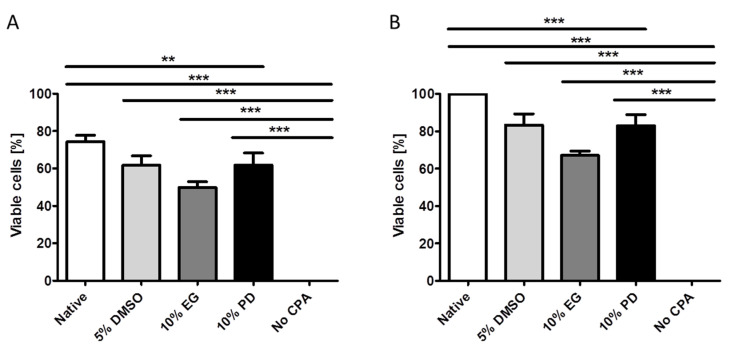
Viability of cell populations containing iPSC-generated MKs harvested at day 19 of differentiation according to trypan blue exclusion viability test. (**A**) Absolute values of number of viable cells after cryopreservation with 5% DMSO, 10% EG, and 10% PD, as well as viability in the positive (native) and negative (cryopreservation without CPA) controls. (**B**) Relative values of the number of viable cells after cryopreservation with 5% DMSO, 10% EG, 10% PD, and in the absence of CPA, normalized against the native control. The data is presented as mean ± SD. **—significant difference to native control, *p* < 0.01; ***—significant difference to native control, *p* < 0.001.

Survival of iPSC-generated MKs after cryopreservation was first assessed using the trypan blue exclusion viability test. The cell population containing iPSC-generated MKs was harvested at day 19 of in vitro differentiation. Among the tested concentrations of the studied CPAs, maximal survival was achieved with the use of cryopreservation medium containing 5% DMSO, 10% EG, and 10% PD, which resulted in 61.75% ± 9.88%, 49.75% ± 6.18%, and 61.75% ± 12.87% survival, respectively (Figure 1A), with 10% EG containing medium providint significantly lower survival rates in comparison to the positive control (*p* < 0.01). These optimal CPA concentrations were used in the further experiments. As expected, no viable cells were detected in the negative control samples after cryopreservation in the absence of CPA. Only the fragmented fractions of bursted cells stained with trypan blue were observed. Therefore, due to the complete lack of survival, negative control samples were not evaluated in the following morphological and functional characterization after cryopreservation. Cryopreservation with 10% DMSO showed statistically similar survival compared to cryopreservation with 5% DMSO (data not shown). The lower concentration was selected for the further experiments since it is generally preferred to reduce the DMSO concentration in cryopreservation media due to cytotoxicity [31,32,33,34,35,36,56]. Importantly, it was observed, that even the native (non-cryopreserved) cells harvested from the MK differentiation cultures had no more than 74.25% ± 6.95% of viable cells. This is explained by the peculiarities of mature MK membranes as well as by the necessary handling procedures of the total cell population during MK differentiation. Therefore, the viability outcome after cryopreservation was normalized to the native cell input (Figure 1B). In this case, the level of survival reached 83.31 ± 12.04% after cryopreservation with 5% DMSO, 66.99% ± 4.83% after cryopreservation with 10% EG, and 82.85 ± 12.01% after cryopreservation with 10% PD, resulting in significant reduction of survival after cryopreservation with 10% EG containing cryopreservation medium (*p* < 0.001). 

Since the trypan blue exclusion viability test is only based on membrane integrity, it does not allow revealing the underlying mechanisms that may influence cell viability [57]. Therefore, in order to detect and distinguish necrotic and apoptotic processes, which may have been triggered during cryopreservation procedures, cell viability was further evaluated using Annexin V and PI staining.

**Figure 2:**
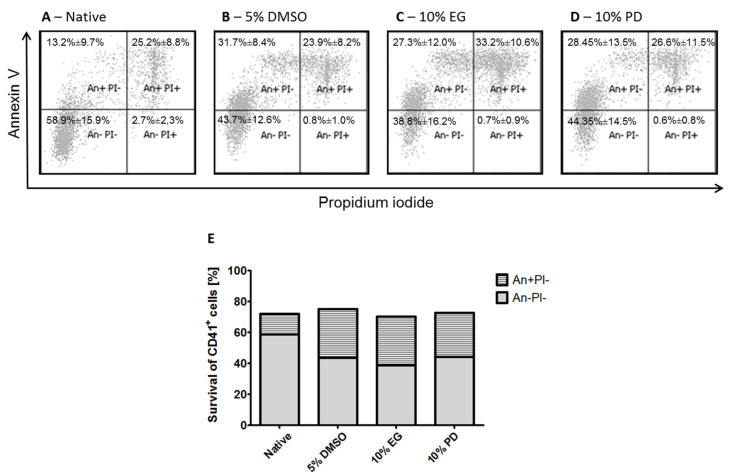
Levels of necrosis and apoptosis in the in vitro iPSC-derived MK cell populations before and after cryopreservation. Staining with Annexin V and PI analyzed with flow cytometry. MKs stained with anti-CD41 antibody. Representative dot plots before (**A**) and after ((**B**)—5% DMSO; (**C**)—10% EG; (**D**)—10% PD) cryopreservation. The data is presented as mean ± SD. (**E**)—Total percentages of MKs with potential contribution to the cell survival. The data is presented as a sum of means of MK cells detected negative for both necrotic and apoptotic events with means of MK cells negative for necrotic, but positive for apoptotic events.

Cells that are only stained with Annexin V are at the early stage of apoptosis, whereas cells that are also stained with PI undergo necrosis as well as late apoptosis processes. Additionally, the cells were stained with anti-CD41 antibody in order to identify the MKs with typical phenotype in the whole cell population. It was found that cells cryopreserved with 5% DMSO showed 43.7% ± 12.6% of MK population negative for both necrotic and apoptotic events (Annexin^−^, PI^−^), while 31.7% ± 8.4% of the cells were negative for necrotic events, but positive for apoptotic events (Annexin^+^, PI^−^) (Figure 2B). Cells cryopreserved with 10% EG showed 38.8% ± 16.2% of MK population negative for both necrotic and apoptotic events, while 27.3% ± 12.0% of cells were negative for necrotic events and positive for apoptotic events (Figure 2C). Finally, the cells cryopreserved with 10% PD showed 44.35% ± 14.5% of MK population negative for both necrotic and apoptotic events and 28.45% ± 13.5% of cells were negative for necrotic events, but positive for apoptotic (Figure 2D). Similarly to trypan blue exclusion studies, native (non-cryopreserved) cells harvested from the differentiation cultures were also tested with Annexin V/PI staining and it was found that only 58.9% ± 15.9% of MKs in the whole cell population were negative for both necrotic and apoptotic events and as much as 13.2% ± 9.7% were negative for necrotic events, but apoptotic even before cryopreservation (Figure 2A). It should be noted that necrotic processes are usually irreversible, whereas cells may still be capable to recover from apoptosis with the help of intracellular reparative mechanisms, thus contributing to the terminal cell survival [58,59]. Annexin V binds to phosphatidylserine, which is normally found on the intracellular leaflet of the plasma membrane of healthy cells. However, Annexin V has also been shown to bind to certain viable non-apoptotic blood cells [60]. In particular, due to the specific functional properties of MKs to produce PLTs through a number of remodeling events occurring in membrane and cytoplasm, some Annexin V may also bind to viable MKs possessing functional intrinsic apoptosis pathway, not related to cryopreservation damage [8,53,61,62]. Considering the possibility that some cells that were detected positive for apoptotic events but negative for necrotic markers could actually contribute to the surviving MK population after recovery from cryopreservation, the established protocols resulted in acceptable cryosurvival rates (Figure 2E). Moreover, under these circumstances the data correspond well with the survival rates determined based on the conventional primary evaluation with trypan blue staining.

**Figure 3:**
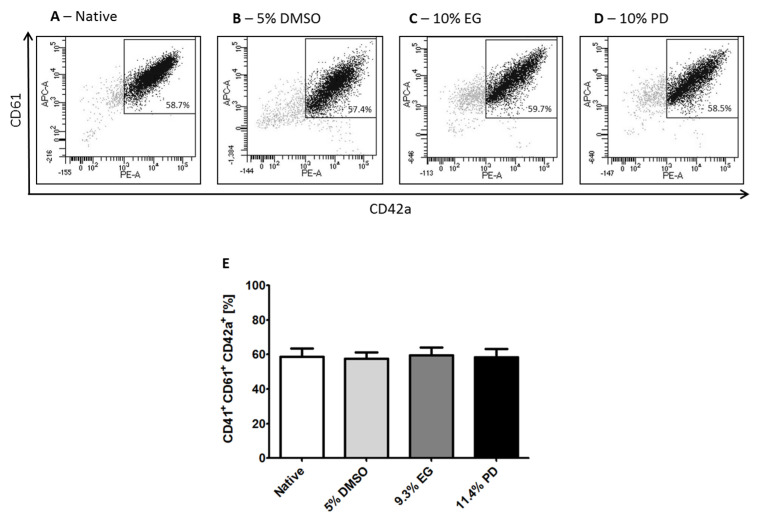
Frequencies of MK Population of MKs before and after cryopreservation. Differentiated MKs were identified by FSC and SSC properties and by the expression of CD41, CD42a, and CD61 markers. Representative dot plots of the flow cytometric analysis of population of MKs in the cells harvested from iPSC differentiation. Dot plots represent CD42a and CD61 double positive population gated from the CD41 positive population. Samples before (**A**) and after ((**B**)—5% DMSO; (**C**)—10% EG; (**D**)—10% PD) cryopreservation. (**E**)—Percentage of triple positive MKs from the total cell population at the terminal phase of differentiation, represented as mean ± SD.

The pattern of molecules expressed on the cell surface is not only necessary for their characterization, but also crucial for the proper cell type-specific function. At day 19 of iPSCs differentiation 58.7% ± 9.6% of the harvested cell population was triple positive for CD61, CD41, and CD42a MK markers (Figure 3A). After recovery from the low temperature storage, the cells cryopreserved with 5% DMSO had 57.4% ± 7.5% of triple positive MKs, as well as 59.7% ± 8.7% in the case of cryopreservation with 10% EG, and 58.5% ± 9.2% when cryopreserved with 10% PD (Figure 3B–D).

**Figure 4:**
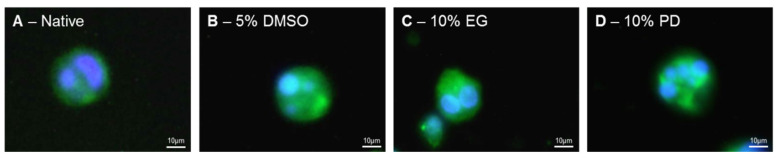
Polyploidy of in vitro iPSC-derived MKs before (**A**) and after ((**B**)—5% DMSO; (**C**)—10% EG; (**D**)—10% PD) cryopreservation. Representative fluorescence microscopy image of MKs stained with anti CD61-FITC antibody (green), nuclei stained with DAPI (blue).

Our data shows that cryopreservation with 5% DMSO, 10% EG, and 10% PD allows preserving expression of the typical phenotypic MK markers at the levels statistically similar to the native samples. Inherent to MKs polyploidy was evaluated with two different approaches. First, visualization of the multiple nuclei with fluorescence microscopy allowed observing that the MK populations surviving applied cryopreservation procedures maintain their polyploidy features similar to the cells before cryopreservation (Figure 4).

**Figure 5:**
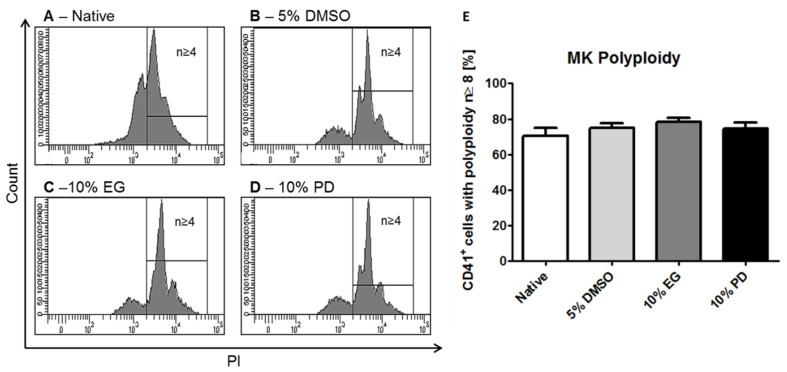
Polyploidy of in vitro iPSC-derived MKs before (**A**) and after ((**B**)—5% DMSO; (**C**)—10% EG; (**D**)—10% PD) cryopreservation. Propidium iodide staining analyzed with flow cytometry. MKs stained with anti-CD41 antibody. Representative histograms with gated ploidy *n* ≥ 4. (**E**)—Percentage of MKs with ploidy *n* ≥ 4, represented as mean ± SD.

In addition, flow cytometric analysis confirmed these observations, showing that iPSC-derived MKs possess an increased DNA content at the studied stage of maturation (Figure 5). On day 19 of differentiation 70.4% ± 8.8% of MK cell population exhibited DNA contents of *n* ≥ 4. Applied cryopreservation procedures resulted in recovery of MKs with the ploidy of *n* ≥ 4 on the levels of 74.9% ± 6.0% in the case of low temperature storage with 5% DMSO, 78.4% ± 5.2% when frozen with 10% EG, and 74.5% ± 7.6% in the case of 10% PD use. Therefore, these CPAs under studied concentrations and cooling rate conditions allow preserving polyploid populations of MKs on the levels statistically similar to the initial before freezing.

### 2.2. Functional Activity of MKs after Cryopreservation

**Figure 6:**
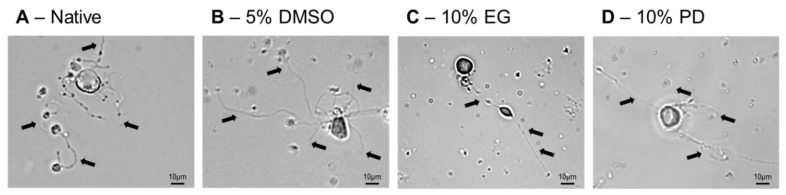
Functional capability of in vitro iPSC-derived MKs to form proPLTs before (**A**) and after ((**B**)—5% DMSO; (**C**)—10% EG; (**D**)—10% PD) cryopreservation. ProPLTs indicated with arrows.

Further to the basic parameters of survival after cryopreservation, such as the analysis of membrane integrity and intensity of apoptotic and necrotic events, as well as after comprehensive phenotypic characterization, it is crucial to investigate the recovery of functional properties of the cells after the impact of subzero temperatures. Already after 24 h of recovery after cryopreservation the cells were capable to generate proPLTs similar to the cells prior freezing (Figure 6). The most pronounced visual similarities of the formed proPLTs after low temperature storage to the non-cryopreserved MKs were observed in the case of freezing with 5% DMSO and 10% PD (Figure 6A,B).

**Figure 7:**
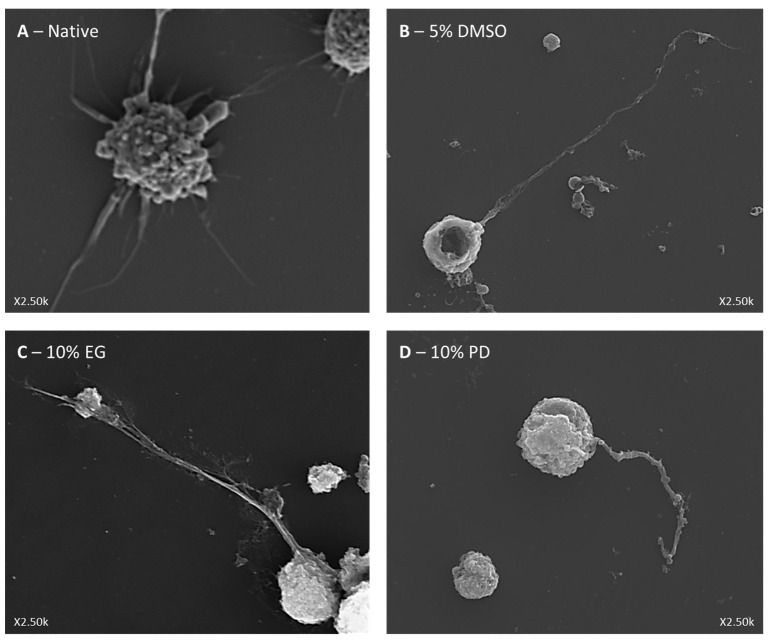
Ultrastructural analysis of proPLT formation by in vitro iPSC-derived MKs before (**A**) and after ((**B**)—5% DMSO; (**C**)—10% EG; (**D**)—10% PD) cryopreservation. Magnification ×2.5k.

Additionally, we performed ultrastructural analysis of proPLT formation before and after cryopreservation with scanning electron microscopy (Figure 7). We were able to observe the capability of proPLT formation in all studied samples with unaltered ultrastructural morphology.

**Figure 8:**
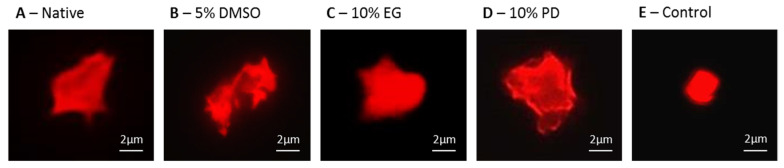
Functional PLTs activated with ADP and thrombin are capable to adhere to fibrinogen. Phalloidin-Texas Red staining binds to actin, allowing visualization of cytoskeleton. PLTs released from in vitro iPSC-derived MKs before (**A**,**E**) and after ((**B**)—5% DMSO; (**C**)—10% EG; (**D**)—10% PD) cryopreservation. (**E**) nonstimulated control.

Furthermore, we assessed the functional capability of PLTs, released by native and cryopreserved iPSC-derived MKs, to activation in response of physiological stimulants ADP and thrombin. A staining with phalloidin, which binds to actin, allowed visualizing the cytoskeleton of the studied PLTs. Capability of PLTs to activate upon stimulation was confirmed under all of the studied cryopreservation conditions as well as in the native samples (Figure 8A–D). PLTs in the negative control, in the absence of ADP and thrombin stimulation, did not activate the spreading capacity (Figure 8E).

### 2.3. In Vivo Transfusion of iPSC-Derived MKs after Cryopreservation

**Figure 9:**
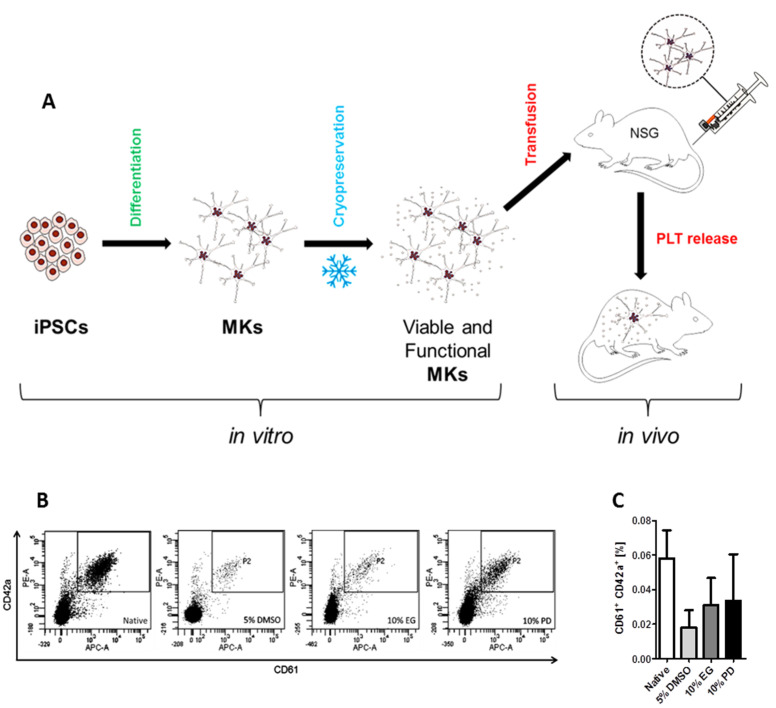
Cryopreserved in vitro iPSC-derived MKs produce PLTs after transfusion into a mouse model. Mice were transfused with 3 × 10^6^ iPSC-derived MKs before and after cryopreservation by tail vein injection. Frequencies of human PLTs were detected 1 h after MK infusion by flow cytometry analysis of the peripheral blood of mice. (**A**) Schematic representation of the experiment: in vitro—differentiation of iPSCs into mature MKs, cryopreservation, recovery of viable and functional MKs after cryopreservation; in vivo—transfusion into mouse model, PLT production in the blood stream. (**B**) Representative dot plots of the flow cytometric analysis of population of human PLTs released to the blood stream of a mouse 1 h after transfusion of cryopreserved and native MKs. (**C**) The graph shows frequencies of the human PLTs detected after MK transfusion in three independent experiments for each sample presented as mean ± SD, *n* = 3.

To support our in vitro results indicating on successful survival of analyzed cryopreservation procedures by the iPSC-derived MKs in a functional form we performed transfusion of these cells before and after cryopreservation procedures to NOD/SCID/IL-2Rγc^–/–^ mice (Figure 9A). Already after 1 h post transfusion we were able to detect human PLTs in the murine circulation in mice transfused with the non-cryopreserved MKs as well as in mice transfused with the MKs after cryopreservation with 5% DMSO, 10% EG, and 10% PD (Figure 9B,C). While the tendency to release PLTs to blood circulation was the most prominent in the samples after cryopreservation with 10% PD and the least expressed in the samples after cryopreservation with 5% DMSO in comparison to the non-cryopreserved MKs, statistical calculations did not reveal significant differences.

Additionally, it was necessary to track the fate of MKs post transfusion in recipient’s organism. Biodistribution assays were performed after 1 and 14 days post transfusion. No human MKs were detected in spleen, lung, heart, and bone marrow tissues (data not shown) indicating on remaining of transfused MKs in the blood circulation for production of PLTs without further integration in the other systems.

## 3. Discussion

Though, advances in development of innovative therapies based on ex vivo-derived MKs promise alternatives to the challenged donor PLT transfusion, practical implementation in clinics requires readily available off-the-shelf supplies of functional MKs in transfusion-sufficient quantities. Hence, translation into clinical application will be associated with the need to develop robust biobanking technologies. Efficient biobanking strategies would be cost and time-saving by reducing the need for a continuously MK/PLT production and associated quality control and management, and, most importantly, provide a standardized MK supply in a ready-to-use manner.

Among the obligatory requirements to clinically relevant biobanking the key role belongs to the compatibility with good manufacturing practice and good laboratory practice standards, which assumes application of serum- and xeno-free protocols throughout the process. Besides, serious clinical safety concerns are currently being associated with the use of DMSO during cryopreservation [31,32,33,34,35,36]. Therefore, these issues were particularly addressed as the core of this study.

Previously, we developed efficient biobanking technologies for mesenchymal stem cells, blood cells, as well as tissues and tissue engineered constructs [26,32,35,63,64]. Results of these studies show substantial differences in cryopreservation outcomes and necessity to optimize the process for each particular object. Therefore, in this study we utilized our expertise in generating MKs in vitro with our experience in cryobiology in order to develop approaches towards efficient cryobanking of iPSC-derived MKs to support preservation of vitality and functionality after thawing [14,19,30].

Though numerous studies aim at elimination or reduction of the contents of DMSO in cryopreservation medium due to expressed cytotoxicity, it is still the most efficient and widely used CPA [31,32,36,56]. Nevertheless, while majority of conventional cryopreservation protocols require 10% concentration of DMSO in the cryopreservation medium (*vol/vol%*) [31,36], we were recently able to successfully cryopreserve iPSC-derived MKs with DMSO concentration reduced to 7.5% [30]. In the current study we were able to further reduce this concentration by two-fold from the conventional 10% approach. Other reports show, that similar reduction of DMSO concentration to 5% results in decreased side effects in patients, as well as preserved or improved structural and functional parameters after cryopreservation of a range of other cell types, including neutrophils, leucocytes, and hematopoietic stem cells, in comparison to 10% DMSO [65,66,67,68].

Several successful attempts to cryopreserve ex-vivo-derived MKs have been shown. In particular, one group also cryopreserved MKs with the use of 5% DMSO as a CPA [13]. However, applied cryopreservation medium was based on Iscove’s Modified Dulbecco’s Medium (IMDM) supplemented with 20% fetal bovine serum, whereas our cryopreservation conditions, presented in this study, are completely defined and xeno-free. Other researchers have used commercially available CryoStor medium, which contains 10% DMSO and is xeno- and serum-free [29]. Yet, here the premature MK progenitors were cryopreserved, which could be potentially less challenging due to a less complex state of the membrane in comparison to mature MKs, and would require subsequent maturation of MK progenitors after thawing prior to potential application in a patient. The 10% DMSO-based CryoStor medium also provided sufficient recovery after cryopreservation for MKs generated from CD34+ umbilical cord blood cells [69]. In this case, MK generation approach is donor dependent and limited to proliferation capacity of primary umbilical cord cells. Furthermore, other studies show that certain subpopulations of MK cells are highly sensitive to subzero temperature stress resulting in low survival rates even in the donor-derived MKs [27,28]. Even more controversial results apply to biobanking of PLTs. Several attempts towards low temperature storage of PLTs are known already for decades [48,50,70,71]. However, due to the high sensitivity of PLTs to manipulations, significantly reduced recovery and functionality is observed when PLTs are stored hypothermically (4 °C) or cryopreserved [50]. Though transfusion of cryopreserved PLTs have been shown in experimental clinical trials already in the late 1970s, up to now cryopreserved PLTs are not routinely applied in transfusion medicine practice [71]. Thus, in terms of biobanking strategy for clinical application, it seems more feasible to focus on rather cryopreserving MKs or MK-precursors, then the PLTs per se [7,13,69].

Additionally, here we explored the potential of alternative to DMSO CPAs, chosen on the basis of previous data on cryopreservation efficiency, membrane permeability, and levels of cytotoxicity on the other cell types [23,25,26,31,36]. In particular, we were able to achieve highly efficient outcome in preserving viability, phenotype, and functionality of MKs with application of EG and PD. To our knowledge, possibility to cryopreserve iPSC-derived MKs with EG and PD have not been shown in the other studies. Both of these compounds are considered significantly less toxic then DMSO, are approved by FDA, and are routinely applied in pharmacy as well as in cryopreservation of a range of biological specimens [38,39,40,41,42,43,44]. In this work, we assess the cell viability after cryopreservation not only with the conventional trypan blue exclusion test, which is based on the cellular membrane integrity, but also with flow cytometric approaches allowing distinguishing necrotic and apoptotic processes in the cells [57]. Importantly, we normalized our findings on survival of MKs after cryopreservation to the non-cryopreserved control, since membrane remodeling naturally inherent to the functional MKs may influence the outcome results in these tests [53]. We also show, that cryopreservation of iPSC-derived MKs with proposed concentrations of DMSO, EG and PD allows preserving populations expressing typical MK markers and possessing inherent to MK polyploidy on the levels statistically similar to these cells prior freezing. Besides providing the adequate levels of viability and phenotype preservation, successful biobanking technologies assume the possibility of recovery of desired functional parameters. Mature functional MKs should be able to produce PLTs [4,53]. Here we demonstrate that our cryopreservation protocols with application of 5% DMSO, 10% EG, or 10% PD preserve the capability of the ex vivo-derived MKs to form proPLTs and release PLTs after thawing similarly to the non-cryopreserved samples. Ultrastructural analysis of proPLT formation was additionally evaluated with application of scanning electron microscopy. While it was challenging to handle the fragile proPLTs during multiple steps of sample preparation, we were able to observe the capability of MKs to proPLT formation after cryopreservation with selected parameters. PLTs released by the MKs before as well as after the impact of subzero temperatures demonstrated the capability to adhere upon stimulation, which is among the key functional properties necessary in recognition of injuries and blood coagulation [54,55]. Finally, an in vivo mouse model was used to confirm that cryopreserved in vitro-generated MKs are functional after transfusion with further capability to release PLTs into the blood stream. Though non-cryopreserved ex vivo-derived MKs of various origins were previously reported to possess such capability by us and the other groups [19,20,22], development of efficient biobanking technologies allowing preservation of such cells and their functions under defined xeno-free conditions and with reduced or abolished DMSO is novel, highly essential, and may contribute in supporting their pathway into clinical application.

## 4. Materials and Methods

### 4.1. Experimental Design

MKs were generated via differentiation of human iPSCs, as described in the corresponding section. At the terminal stage of differentiation the MKs were harvested from the adherent monolayer and further cultured as suspension cell culture in the APEL medium (STEMCELL Technologies, Vancouver, BC, Canada) supplemented with cytokines equivalent to the terminal stage of differentiation. Afterwards, the MKs were subjected to cryopreservation in the culture medium containing a range of CPAs in different concentrations, including conventional DMSO and alternative EG and PD. The samples were recovered by rapid thawing with subsequent removal of CPA and compared to the native (non-cryopreserved) MKs as a positive control. MKs cryopreserved without CPA served as a negative control. Viability, morphology, and functional characteristics of iPSC-derived MKs were evaluated before and after cryopreservation. Viability was analyzed directly after recovery from cryopreservation with conventional trypan blue exclusion test, as well as with Annexin V and PI staining, which allows detecting necrotic and apoptotic processes. Morphology and functional characteristics were analyzed 24 h after recovery from cryopreservation. Expression of typical MK markers, as well as inherent polyploidy, were analyzed with flow cytometry and fluorescence microscopy. Capability to form proPLTs and release functional PLTs was analyzed with phase-contrast microscopy, fluorescence microscopy, as well as scanning electron microscopy. Furthermore, capability of MKs to release PLTs before and after cryopreservation was evaluated in vivo in a mouse model. Three independent experiments in triplicates were performed for a mouse model and at least five independent experiments in triplicates for in vitro studies.

### 4.2. iPSCs Culture

Human iPSC line hCBiPSC2 was derived from the human cord blood endothelial cells, as described before [72]. In brief, iPSCs were cultured under xeno-free and feeder-free conditions. In brief, 12 well non-tissue culture-treated plates (Falcon™, Corning, Corning, NY, USA) were coated with human recombinant Laminin-521 (BioLamina, Sundbyberg, Sweden), followed by seeding of the cells in 50,000 cells/cm^2^ density and culturing under sterile conditions in StemMACS medium (Miltenyi Biotec, Bergisch Gladbach, Germany) at 5% CO_2_ and 37 °C in humidified CO_2_ incubator (Thermo Fisher Scientific, Waltham, MA, USA) with a daily medium change. Harvesting of the cells was performed with application of TrypLE™ Express Enzyme solution (Life Technologies, Carlsbad, CA, USA).

### 4.3. Generation of MKs and PLTs from Human iPSCs

Human iPSCs were differentiated into MKs by culturing in the APEL medium supplemented with consecutive range of cytokines and growth factors (PeproTech, Rocky Hill, NJ, USA), as described before [19]. IPSCs were cultured on Laminin-521-coated plates in StemMACS medium until approximately 50% confluence, which represented day 0 of differentiation. On day 0, the StemMACS medium was replaced with 1:1 (*vol/vol%*) mixture of StemMACS and APEL medium supplemented with 50 ng/mL of bone morphogenetic protein 4 (BMP4) and 50 ng/mL of vascular endothelial growth factor (VEGF). After 48 h this medium was replaced completely with pure APEL medium supplemented with 50 ng/mL of BMP4 and 50 ng/mL of VEGF. At day 4 of differentiation, the culture medium was replaced with APEL medium supplemented with 50 ng/mL of thrombopoietin (TPO), 50 ng/mL of stem cell factor (SCF) and 25 ng/mL of interleukin-3 (IL-3). Starting from differentiation day 12, the cells were cultured in APEL medium supplemented with 50 ng/mL of TPO and 50 ng/mL of SCF. At day 19 supernatants of the cell culture were collected by gently rinsing the monolayer and harvesting contained detached cells. The supernatants were separated in two fractions with subsequent centrifugation steps and analyzed for the presence of MKs and PLTs. In the first step the supernatant with harvested cells was centrifuged for 10 min at 120× *g* in order to pelletize larger cells which correspond to the estimated size of MKs. Remaining supernatant was collected for centrifugation in the second step, performed for 10 min at 740× *g*. This step allows pelleting smaller cells and cellular fragments, which correspond to the size of PLTs. Fractions of both centrifugation steps were analyzed independently for MK and PLT origin and functionality with morphological and functional tests, which are described below.

### 4.4. Cryopreservation of MKs

Cells present in the culture supernatants at day 19 of differentiation were harvested by gently rinsing the monolayer and centrifugation for 10 min at 120× *g* to pellet the MKs. The MKs were equilibrated in cryoprotective medium at a concentration of 1 × 10^6^ cells/mL at 20 °C for 15 min. Three different CPAs, including DMSO, EG, and PD in volume fractions of 5%, 10%, 15%, and 20% (*vol/vol%*) concentration in APEL medium were tested for their cryoprotective activity. Cryoprotective solutions were added in a dropwise fashion to the cell suspension. The samples were equilibrated with CPA containing medium for 10 min, transferred to 1.8 mL cryovials (Nunc™, Thermo Fisher Scientific) and placed in a Mr. Frosty™ container (Thermo Fisher Scientific) to cool down the samples to −80 °C in a −80 °C freezer at a cooling rate of 1 K min^−1^, followed by immersion of the samples in liquid nitrogen and storage in standard cassette boxes. Rapid thawing of the samples was performed in a water bath at 37 °C with subsequent washing with a culture medium for removing of CPA by centrifugation for 10 min at 120× *g*.

### 4.5. Evaluation of Viability of MKs after Cryopreservation

The viability of cells was determined by staining with 0.4% trypan blue. The total number of cells and the number of cells stained with trypan blue was counted in a Neubauer hemacytometer (Marienfeld-Superior, Lauda-Königshofen, Germany). Percentage of survival was calculated by the formula:

(1) $$\%\;\mathrm{Viable}\;\mathrm{cells}\;=\frac{\mathrm{Number}\;\mathrm{of}\;\mathrm{viable}\;\mathrm{cells}}{\mathrm{Total}\;\mathrm{number}\;\mathrm{of}\;\mathrm{cells}}\times 100$$

Native (fresh, non-cryopreserved) cells from the cell culture were used as a positive control; cells cryopreserved without the CPA were used as a negative control.

In order to detect and distinguish necrotic and apoptotic processes, which may have been initiated in the cells by cryopreservation procedures, the cells were analyzed with application of APC Annexin V Apoptosis detection kit with PI (BioLegend, San Diego, CA, USA) according to the manufacturer’s protocol. For that, the cells were washed twice with cold BioLegend Cell Staining Buffer, and then resuspended in 100 µL of Annexin V Binding Buffer. Then 5 µL of APC Annexin V and 10 µL of propidium Iodide (PI) solution were added. Additionally, the cells were stained with APC-Cy7-conjugated anti-CD41 (GPIIb; BioLegend) antibody in concentrations recommended by the manufacturer in order to detect MKs in the population. The cells were incubated for 15 min at room temperature (24 °C) in the dark. Then 400 µL of Annexin V Binding Buffer were added. Flow cytometry was performed with a FACSCanto™ flow cytometry system (BD Biosciences, San Jose, CA, USA) counting 10,000 events per measurement. The kit is specifically designed for the identification of apoptotic and necrotic cells.

### 4.6. Morphofunctional Characterization of MKs and PLTs before and after Cryopreservation

All morphological and functional tests were performed with MKs and PLTs before and after cryopreservation, in order to determine efficiency of the applied cryopreservation protocols and the influence of low temperatures on phenotype and function.

#### 4.6.1. Immunophenotyping of MKs

Immunophenotyping of iPSC-derived MKs was performed by flow cytometry using a FACSCanto™ flow cytometry system and BD FACSDiva™ software (Becton Dickinson, Franklin Lakes, NJ, USA), as described before [19]. The cells were stained with allophycocyanin (APC)-cyanine 7 (Cy7)-conjugated anti-CD41 (GPIIb; BioLegend), APC-conjugated anti-CD61 (GPIIIa; BioLegend) and phycoerythrin (PE)-conjugated anti-CD42a (GPIX; BD Biosciences) antibodies in concentrations recommended by the manufacturer. Corresponding isotype antibodies were used as controls to exclude the unspecific binding artefact.

#### 4.6.2. Polyploidy of MKs

Polyploidy of differentiated MKs was analyzed by immunocytochemistry. Cells were stained with fluorescein isothiocyanate (FITC)-conjugated anti-CD61 (GPIIIa; BD Biosciences) antibody in concentrations recommended by the manufacturer in order to detect the MKs in population, followed by staining with 4′,6-diamidino-2-phenylindole dihydrochloride nucleic acid stain (DAPI; Invitrogen, Carlsbad, CA, USA). Cell images were acquired by fluorescence microscopy using an Olympus IX81 microscope (Olympus, Shinjuku, Japan) and the Xcellence Pro image software (Olympus).

Frequencies of polyploid MK cells in differentiated cell populations were quantified with flow cytometry. MKs were stained with APC-Cy7-conjugated anti-CD41 antibody (GPIIb; BioLegend), washed with phosphate-buffered saline (PBS) and treated with 200 µL of Cytofix/Cytoperm^TM^ fixation and permeabilization solution (BD Biosciences) for 20 min at room temperature, according to the manufacturer’s protocol. Afterwards, the cells were stained with PI staining solution (10 μg/mL) supplemented with 10 U/mL of RNAse A (Sigma-Aldrich, St. Louis, MO, USA) and acquired with a FACSCanto™ flow cytometry system.

#### 4.6.3. ProPLT Formation

Capability of MKs to form proPLT was studied as a functional parameter of iPSC-derived MKs, as previously described [14,19]. Cells harvested at day 19 of differentiation, or cells directly after cryopreservation, were seeded on 12-well suspension culture plates (Greiner CELLSTAR^®^; Greiner Bio-One, Kremsmünster, Austria) in APEL medium supplemented with 50 ng/mL of TPO and 50 ng/mL of SCF. After 24 h the images of MKs forming proPLTs were acquired using an Olympus IX81 microscope with a digital B/W camera (Olympus) and analyzed with the Xcellence Pro image software. ProPLTs were detected as elongated uniform tubular structures with periodic PLT-sized swellings [73].

#### 4.6.4. Analysis of Ultrastructure with Scanning Electron Microscopy

Scanning electron microscopy was conducted in order to analyze ultrastructure of MKs releasing proPLTs before cryopreservation and after thawing. The cells were centrifuged at 1000× *g* rpm for 5 min and diluted in PBS to yield the final concentration of approximately 1 × 10^6^ cells in 100 µL. Afterwards, 100 µL of the cell suspension was added on top of 12 mm glass cover slips (Carl Roth, Karlsruhe, Germany) previously coated with poly-l-lysine (PLL). The PLL-coated cover slips were prepared by incubating in 0.1% PLL solution prepared in PBS from PLL-hydrobromide (Sigma-Aldrich) at 4 °C overnight, rinsing in distilled water two times and further air drying for 2 h. After incubation of cells on cover slips for 1 h at room temperature, the samples were carefully rinsed twice with 0.1 M cacodylate buffer (CAC, pH 7.4, Carl Roth) in 12-well plates (TPP, Trasadingen, Switzerland) and fixed with 2.5% glutaraldehyde (Carl Roth) in 0.1 M CAC for 60 min. After removal of the fixation solution, the prepared cover slips were washed twice with 0.1 M CAC and distilled water (5 min each step) and dehydrated in a series of ethanol concentrations (25%, 50%, 75%, 90%, 100%) for 10 min each step. After final dehydration in 100% ethanol for 10 min, the prepared cover slips were transported into 12-well plates containing cuts of ashless filter paper (Macherey-Nagel, Carl Roth) and air-dried overnight under the hood. For SEM, the prepared cover slips were fixed on SEM stubs using conductive double-sided glue tape (Plano GmbH, Wetzlar, Germany). In order to avoid charge accumulation during SEM imaging on the surface of the glass cover slips, ACHESON silver painting (Plano GmbH) was carefully applied on the perimeter of the cover slips using a brush. After being air-dried for 60 min, the prepared cover slips were sputter coated twice with gold-palladium for 15 s and observed under high vacuum at 15 kV accelerating high voltage and 5 mm working distance using a scanning electron microscope (S3400N, Hitachi, Tokyo, Japan).

#### 4.6.5. PLT Adherence Assay

PLT adherence glass assay was used to evaluate the functionality of PLTs produced by iPSC-derived MKs, as described before [14]. In brief, the PLTs were separated from MKs as described above and stimulated for 90 min at 37 °C with 1 mmol/L of adenosine 5′-diphosphate and 1 U/mL of thrombin on fibrinogen coated PCA plates (Sarstedt, Nümbrecht, Germany). Afterwards, the cells were washed with PBS and fixed for 10 min with BD Cytofix™ Fixation Buffer (BD Biosciences). Then the cells were washed twice with 100 mM glycine in PBS and once with pure PBS. Washing was followed by incubation with 0.2% Triton-X-100 (Sigma-Aldrich) in PBS for permeabilization of cellular membranes and staining with phalloidin-TexasRed (Invitrogen). After washing with PBS and H_2_O the grid of the PCA plates was detached and the slide was covered dropwise with the mounting solution supplemented with DAPI fluorescent stain (Dianova, Hamburg, Germany). Then the slide was covered with the coverslip and dried overnight at room temperature. Next day the slides were analyzed for adherence of the activated PLTs to the fibrinogen covered surface with application of fluorescence microscopy using the TxRed and the DAPI channel. Visualized PLTs were differentiated from MKs in the overlay pictures based on cell size and the absence of a nucleus. Nonstimulated PLTs served as a negative control.

### 4.7. In Vivo PLT Transfusion Refractoriness Mouse Model

A mouse model was used to analyze the capability of fresh and cryopreserved in vitro iPSC-derived MKs to release PLTs in vivo. 3 × 10^6^ MKs were transfused by intravenous injection into 8 to 12 week old NOD/SCID/IL-2Rγc^–/–^ mice and allowed to distribute within the blood circulation. Peripheral blood of a mouse was drawn 1 h after transfusion in order to analyze the samples for the presence of human PLTs. Human PLTs were identified by flow cytometry by staining with anti-human PE-conjugated anti-CD42a (GPIX; BD Biosciences) and APC-conjugated anti-CD61 (GPIIIa; BioLegend) antibodies in concentrations recommended by the manufacturer. Experimental mice were maintained under defined pathogen-free conditions in the animal facility of the Helmholtz Centre for Infection Research (Braunschweig, Germany). All animal experiments in this study were performed according to the legislative requirements and ethical standards in agreement with the local government of Lower Saxony (Az: 33.42505-084/06, Germany).

### 4.8. Data Analysis and Statistics

The data was collected from at least 5 independent experiments in triplicates for in vitro studies and from 3 independent experiments in triplicates for in vivo model (*n* ≥ 3). Statistically significant conclusions were obtained by analysis of mean values and standard deviation calculations (mean ± SD) with Mann-Whitney criteria and Fisher’s method. Parameter changes were considered statistically significant at *p* < 0.05 in ANOVA test. Statistical calculations and data analysis were performed with GraphPad Prism software (GraphPad Software, Inc., San Diego, CA, USA).

## 5. Conclusions

In summary, combination of efficient biobanking technologies using defined GMP-compatible procedures with the possibility of large-scale in vitro production of MKs and PLTs provides an indispensable potential for the development of novel cell therapeutic strategies to manage thrombocytopenic patients. In this work, we showed the possibility to successfully cryopreserve bioengineered MKs with application of significantly reduced DMSO concentrations in comparison to conventional suspension cell culture protocols, as well as applied alternative DMSO-free CPA-based protocols with a similar outcome. Besides the eventual clinical perspectives in the field of transfusion medicine, such technologies also allow significant improvement of in vitro PLT production by providing the possibility to store MKs in necessary amounts and of desired quality and state.

## Abbreviations

iPSC	Induced Pluripotent Stem Cell
MK	Megakaryocytes
PLT	Platelets
proPLT	Proplatelets
DMSO	Dimethyl sulfoxide
EG	Ethylene glycol
PD	Propane-1,2-diol
CPA	Cryoprotective agent
GMP	Good manufacturing practice
BMP4	Bone morphogenetic protein 4
VEGF	Vascular endothelial growth factor
SCF	Stem cell factor
IL-3	Interleukin-3
TPO	Thrombopoietin
PBS	Phosphate buffered saline
PLL	Poly-L-lysine
CAC	Cacodylate buffer
DAPI	4′,6-diamidino-2-phenylindole
